# Microbial metabolite butyrate modulates granzyme B in tolerogenic IL-10 producing Th1 cells to regulate intestinal inflammation

**DOI:** 10.1080/19490976.2024.2363020

**Published:** 2024-06-06

**Authors:** Wenjing Yang, Tianming Yu, Xia Liu, Suxia Yao, Kamil Khanipov, George Golovko, Danyvid Olivares-Villagómez, Yingzi Cong

**Affiliations:** a Division of Gastroenterology and Hepatology, Department of Medicine, Feinberg School of Medicine, Northwestern University, Chicago, IL, USA; b Center for Human Immunobiology, Feinberg School of Medicine, Northwestern University, Chicago, IL, USA; c Department of Microbiology and Immunology, University of Texas Medical Branch, Galveston, TX, USA; d Department of Pharmacology, University of Texas Medical Branch, Galveston, TX, USA; e Department of Pathology, Microbiology and Immunology, Vanderbilt University Medical Center, Nashville, TN, USA

**Keywords:** Microbiota, GzmB, colitis, glucose metabolism

## Abstract

CD4^+^ T cells play a critical role in regulating autoimmune diseases, and intestinal microbial metabolites control various immune responses. Granzyme B (GzmB)-producing CD4^+^ T cells have been recently reported to participate in the pathogenesis of autoimmune diseases. Here, we found that GzmbB-deficient CD4^+^ T cells induced more severe colitis in *Rag1*^−/−^ mice than wild-type (WT) CD4^+^ T cells. Germ-free (GF) mice exhibited a lower expression of GzmB in intestinal CD4^+^ T cells compared to specific pathogen-free (SPF) mice. Intestinal microbial metabolite butyrate increased GzmB expression in CD4^+^ T cells, especially in IL-10-producing Th1 cells, through HDAC inhibition and GPR43, but not GPR41 and GPR109a. Butyrate-treated GzmB-deficient CD4^+^ T cells demonstrated more severe colitis compared to butyrate-treated WT CD4^+^ T cells in the T cell transfer model. Butyrate altered intestinal microbiota composition, but altered microbiota did not mediate butyrate induction of intestinal CD4^+^ T cell expression of GzmB in mice. Blimp1 was involved in the butyrate induction of GzmB in IL-10-producing Th1 cells. Glucose metabolism, including glycolysis and pyruvate oxidation, mediated butyrate induction of GzmB in Th1 cells. In addition, we found that IKZF3 and NR2F6 regulated GzmB expression induced by butyrate. Together, our studies underscored the critical role of GzmB in mediating gut bacterial metabolite butyrate regulation of T cell tolerance at the mucosal surface.

## Introduction

Microbiome has been implicated in regulating many health problems and conditions.^1^ Identifying a disease state in which the microbiome has not been involved in scientific literature has become challenging. However, the causal claims in the microbiome field are often overstated and disproportional to the experimental evidence from which they are derived. Functional studies become essential to understanding the role of the microbiome in various conditions and diseases. In addition to gut bacterial products which function as TLR ligands regulating various immune responses, bacterial metabolites have emerged as crucial in maintaining intestinal homeostasis and regulating intestinal inflammation.^2^ Among gut bacterial metabolites, short-chain fatty acids (SCFAs), the gut bacterial metabolites by fermentation of dietary fibers, have been shown to contribute to the maintenance of intestinal homeostasis by regulating various immune cells at the mucosal surface. We and others have shown that SCFAs promote intestinal epithelial tissue repair and enhance intestinal IgA responses to gut microbiota.^3–6^

Accumulating evidence indicates that gut microbiota antigen-specific CD4^+^ T cells are essential in regulating intestinal inflammation and maintaining intestinal homeostasis.^7^ Th1 and Th17 cells mediate intestinal inflammation by producing proinflammatory cytokine IFNγ and IL-17, respectively, whereas Treg cells suppress colitis by producing TGF-β and IL-10. We recently demonstrated that SCFAs regulate intestinal CD4^+^ T cell development and promote Th1 cell production of IL-10 and IL-22 to suppress intestinal inflammation.^8,9^ However, how SCFAs regulate intestinal CD4^+^ T cell development and function is still not completely understood.

Granzyme B (GzmB), a functional hallmark of CD8^+^ T cells and natural killer cells in killing various infected cells and tumor cells, has also been shown to regulate CD4 T cell function.^10^ GzmB is involved in T cell suppression by Treg cells.^11^ A recent report further demonstrated that CD4^+^ T cell expression of GzmB inhibits T cell-mediated colitis by inhibiting Th17 cell development.^12^ However, how T cell expression of GzmB is regulated in the intestine is still unclear. In this report, we demonstrated that GzmB-producing CD4^+^ T cells were decreased in Germ-free (GF) mice, and SCFA butyrate promotes GzmB expression in CD4^+^ T cells, especially IL-10-producing Th1 cells, through HDAC inhibition and GPR43 but not GPR41 and GPR109a, which contributes to butyrate inhibition of colitis development.

## Results

### Butyrate increases CD4^+^ T cell expression of GzmB *in vitro and in vivo*

**Figure 1.**
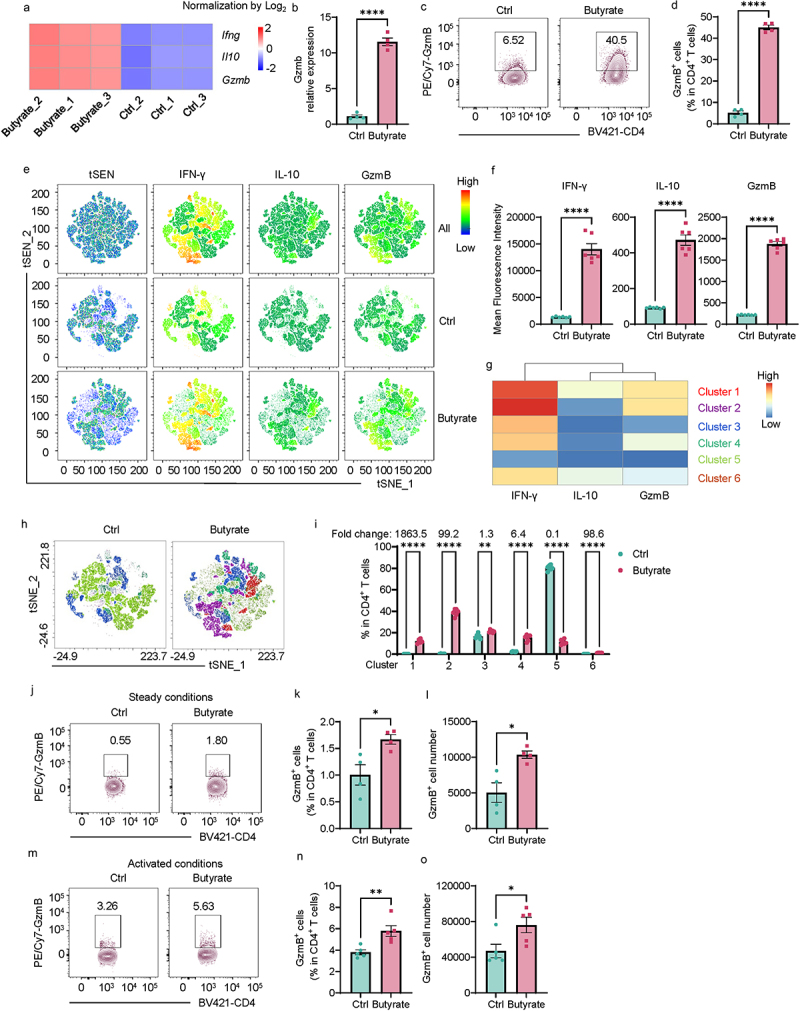
Butyrate increases GzmB expression in Th1 cells.

To investigate the effect of butyrate on gene expression in CD4^+^ T cells, we activated mouse splenic CD4^+^ T cells with anti-CD3 and anti-CD28 antibodies in the presence or absence of butyrate under neutral and Th1 conditions, and gene expression was determined by RNA sequencing.^9^ In addition to IFN-γ and IL-10, which have been reported previously,^13^ we found that butyrate promoted GzmB expression in CD4^+^ T cells under neutral (Supplementary Figure S1A) and Th1 conditions (Figure 1A). GzmB expression was much higher in CD4^+^ T cells under Th1 conditions than cells under neutral conditions (Supplementary Figure S1B), so we used Th1 cells for the following experiments. The granzyme family genes were also determined by RNA sequencing. We found that butyrate increased both GzmB and GzmA expression but did not affect other granzyme family genes, including GzmK, GzmD, GzmM, GzmF, and GzmC in T cells under Th1 conditions (Supplementary Figure S1C), which was confirmed with flow cytometry (Supplementary Figure S1D). However, GzmA expression was much lower than GzmB expression in Th1 cells (Supplementary Figure S1E).

(A-I) Splenic CD4^+^ T cells were activated with anti-mCD3 and anti-mCD28 in the presence or absence of butyrate (500 µM) under Th1 conditions. (A) Cells were collected after 2 days for RNA-Seq (*n* = 3/group). Heatmap of *Ifng*, *Il10*, and *Gzmb* between two groups. Arbitrary units. (B) *Gzmb* mRNA expression in T cells on day 2 (*n* = 4/group). (C) Representative flow cytometry plots of GzmB^+^ CD4^+^ T cells on day 5. (D) Quantification of GzmB^+^ CD4^+^ T cells on day 5 (*n* = 4/group). (E) Representative flow cytometry t-distributed stochastic neighbor embedding (t-SNE) plots of T cells with the expression distribution of IFN-γ, IL-10, and GzmB on day 5. Arbitrary units. (F) Quantification of Mean Fluorescence Intensity of IFN-γ, IL-10, and GzmB in T cells on day 5 (*n* = 6/group). (G) The expression of IFN-γ, IL-10, and GzmB in different clusters on day 5. Arbitrary units. (H) Representative of clusters in tSEN plots on day 5. (I) Quantification of clusters in total T cells on day 5 (*n* = 5/group).

(J–L) WT mice were given normal drinking water or drinking water with butyrate (200 mM) (*n* = 4/group). Mice were killed after two weeks. (J) Representative flow cytometry plots of GzmB^+^ CD4^+^ T cells in the colon. (K) Quantification of GzmB^+^ CD4^+^ T cells in the colon. (L) The absolute number of GzmB^+^ CD4^+^ T cells in the colon.

(M-O) WT mice were given water or drinking water with butyrate (200 mM) (*n* = 5/group) for two weeks and intraperitoneally administered with an anti-CD3 antibody (0.75 mg/kg) twice before being killed. (M) Representative flow cytometry plots of GzmB^+^ CD4^+^ T cells in the colon. (N) Quantification of GzmB^+^ CD4^+^ T cells in the colon. (O) The absolute number of GzmB^+^ CD4^+^ T cells in the colon.

All data are presented as mean ± SEM and are one representative of three independent experiments (B-O). unpaired Student’s *t*-test; *p < 0.05, **p < 0.01, ****p < 0.0001.

Consistent with the RNA sequencing data, butyrate increased GzmB both in mRNA and protein levels determined by qRT-PCR and flow cytometry (Figure 1B–D). To better visualize the potential clusters co-expressing IFN-γ, IL-10, or/and GzmB, we used the t-SNE analysis of CD4^+^ T cells treated with or without butyrate according to the expression of IFN-γ, IL-10, and GzmB (Figure 1E,F) and six different clusters were found (Figure 1G). As shown in Figure 1H,I, clusters 1, 2, 3, 4, and 6 were increased in CD4^+^ T cells after butyrate treatment. Among these clusters, cluster 1, in which CD4^+^ T cells express IFN-γ^high^, IL-10^high^, and GzmB^high^, demonstrated the highest fold change induced by butyrate (1863.5-fold change), followed by cluster 2 (99.2-fold change), in which CD4^+^ T cells express IFN^high^, IL-10^−^, and GzmB^high^. These data suggest that butyrate promotes GzmB expression in both pathogenic IFN-γ^+^ Th1 cells and tolerogenic IL-10^+^ IFN-γ^+^ Th1 cells.

As butyrate constitutes one of the most abundant metabolites produced by the gut microbiome, we then compare the CD4^+^ T cell expression of GzmB in germ-free (GF) mice and specific pathogen-free (SPF) mice. CD4^+^ T cell expression of GzmB and the absolute number of GzmB-producing CD4^+^ T cells were higher in SPF mice compared with GF mice (Supplementary Figure S2A–C). Next, we investigated whether butyrate promotes GzmB expression *in vivo*. We treated wild-type (WT) mice with or without butyrate in drinking water for two weeks, which increased butyrate concentration in the intestine.^9^ Mice treated with butyrate showed a higher percentage and absolute number of GzmB in intestinal CD4^+^ T cells than those without butyrate treatment (Figure 1J–L). We also activated T cells *in vivo* by injection of anti-CD3 antibody into mice pretreated with or without butyrate and found that butyrate increased T cell expression of GzmB and the absolute number of GzmB^+^ T cells in the intestine under activated conditions (Figure 1M–O).

### CD4^+^ T cell expression of GzmB regulates intestinal inflammation

**Figure 2.**
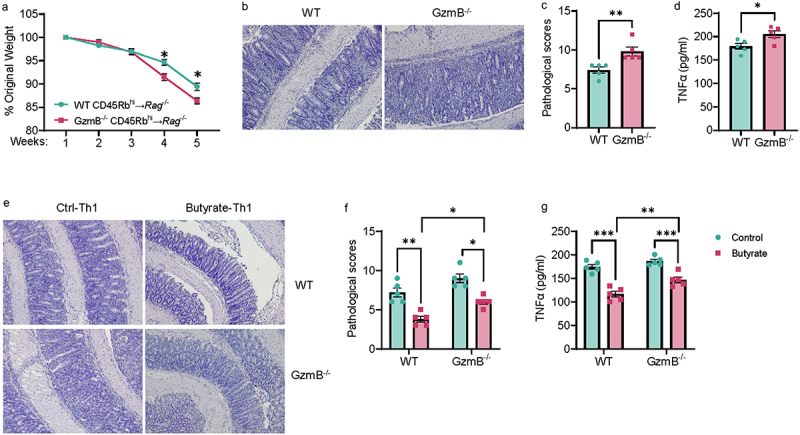
Butyrate-pretreated Th1 cells induced less severe colitis partially through GzmB.

To verify the effect of GzmB in CD4^+^ T cells on intestinal inflammation, we transferred WT and GzmB-deficient CD4^+^ CD45RB^hi^ T cells to immune-deficient *Rag1*^−/−^ mice. When the recipient mice were sacrificed after five weeks post cell transfer, we found that *Rag1*^−/−^ recipients of GzmB-deficient CD4^+^ T cells demonstrated exacerbated colitis compared to recipients of WT T cells, including more weight loss, increased pathological scores, and elevated levels of TNF-α in the intestine (Figure 2A–D). In addition, the number of colonic CD4^+^ T cells was similar in recipients of WT T cells and the recipients of GzmB-deficient T cells (Supplementary Figure S3), indicating that deficiency of GzmB does not affect T cell trafficking to the colon. These data suggest that CD4^+^ T cells derived-GzmB is important in regulating intestinal homeostasis.

(A–D) CD4^+^ CD45Rb^hi^ T cells (1 × 10^5^ cells/mouse) from WT mice or GzmB^−/−^ mice were intravenously transferred to *Rag*^−/−^ mice (*n* = 5/group). Mice were sacrificed five weeks later. (A) Weight change. (B) Representative H&E of recipient *Rag*^−/−^ mice. (C) Pathological scores of recipient *Rag*^−/^. (D) Colonic secretion of TNF-α from recipient *Rag*^−/−^ mice.

(E–G) WT and GzmB^−/−^ CD4^+^ T cells were activated with anti-mCD3 and anti-mCD28 in the presence or absence of butyrate (500 µM) under Th1 polarization conditions. Cells were collected after 5 days and transferred to *Rag*^−/−^ mice (*n* = 5/group). Mice were sacrificed five weeks later. (E) Representative H&E of recipient *Rag*^−/−^ mice. (F) Pathological scores of recipient *Rag*^−/^. (G) Colonic secretion of TNF-α from recipient *Rag*^−/−^ mice.

All data are presented as mean ± SEM and are one representative of two independent experiments. (A, D, and G) unpaired Student’s *t*-test; (C and F) Mann – Whitney U test; *p < 0.05, **p < 0.01, ***p < 0.001.

Next, we transferred control or butyrate-pretreated WT or GzmB-deficient Th1 cells to *Rag1*^−/−^ mice. Consistent with our previous study,^13^ we found that butyrate-pretreated WT Th1 cells induced less severe colitis and lower levels of TNF-α in the intestine in *Rag1*^−/−^ mice than control WT Th1 cells (Figure 2E–G). Although *Rag1*^−/−^ recipients of butyrate-pretreated GzmB^−/−^ Th1 cells demonstrated less severe colitis levels of TNF-α compared to recipients of control GzmB^−/−^ Th1 cells, butyrate-pretreated GzmB^−/−^ Th1 cells induced more severe colitis and elevated levels of TNF-α than butyrate-pre-treated WT Th1 cells (Figure 2E–G). These data indicate that GzmB is involved in the butyrate regulation of Th1 cells’ pathogenicity.

Next, we investigated how GzmB-producing Th1 cells regulate intestinal inflammation. Considering that excessive effector CD4^+^ T cell responses are pivotal in triggering intestinal inflammation, we next investigated whether T cell production of GzmB restrains an exaggerated T cell response by cytotoxic activity. To this end, we co-cultured WT or GzmB^−/−^ Th1 cells with CD45.1 naïve CD4^+^ T cells in the presence of anti-CD3 and anti-CD28 antibodies. After 60 hours, cells were collected and stained with 7-AAD for analysis of cell viability. We found that both WT and GzmB-deficient Th1 cells suppressed the death of CD45.1 cells (Supplementary Figure S4A,B). Additionally, the cell viability of CD45.1 T cells was similar when co-cultured with WT or GzmB-deficient Th1 cells (Supplementary Figure S4A,B). These data suggest that cytotoxic activity may be not the most important function of Th1-expressed GzmB in regulating T cell-mediated diseases. Interestingly, while both WT and GzmB-deficient Th1 cells suppressed CD45.1 T cell proliferation, the capacity to inhibit T cell proliferation was compromised in GzmB-deficient Th1 cells (Supplementary Figure S4C,D).

### Butyrate-altered gut microbiota does not increase CD4^+^ T cell expression of GzmB in mice

**Figure 3.**
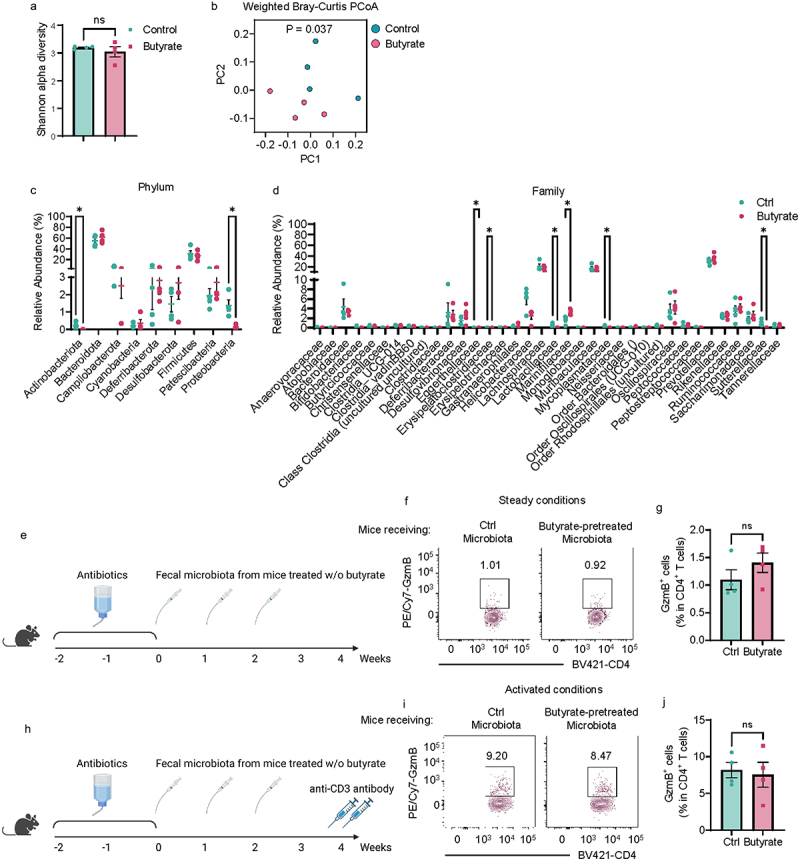
Fecal microbiota from butyrate-pretreated mice does not increase intestinal T-cell production of GzmB.

Gut microbiota, a primary source of butyrate, is important in regulating intestinal responses. Conversely, butyrate has been reported to impact the intestinal microbiota. Therefore, we investigated whether gut microbiota is involved in butyrate induction of GzmB in the gut. First, we found that butyrate treatment did not affect bacterial alpha diversity (Figure 3A), which represents microbiota richness and evenness, but altered microbiota composition, which was determined by Principal Coordinate Analysis (PCoA) (Figure 3B). In addition, *Actinobacteriota* and *Proteobacteria* exhibited decreased abundance at the phylum level in the intestines of mice receiving butyrate (Figure 3C). Moreover, the treatment of butyrate led to a reduction in the abundance of *Eggerthellaceae*, *Erysipelatoclostridiaceae*, *Lactobacillaceae*, *Mycoplasmataceae*, and *Sutterellaceae*, while increasing *Marinifilaceae* at the Family level in the intestine of mice (Figure 3D). To investigate whether butyrate-altered microbiota mediates intestinal CD4^+^ T cell expression of GzmB, we conducted the fecal microbiota transfer experiments in antibiotics-pretreated WT mice, which were treated with or without anti-CD3 antibodies (Figures 3E,H). There was no difference in intestinal T cell expression of GzmB between antibiotics-pretreated mice receiving control gut microbiota and butyrate-pretreated gut microbiota in both steady and activated conditions (Figure 3F–J).

(A–D) WT mice (*n* = 4/group) were treated with or without butyrate in drinking water for two weeks. Feces were collected for 16s rDNA-seq. (A) The Shannon alpha diversity. (B) The Principal Coordinate Analysis (PCoA). (C) Microbiota changes in Phylum level. (D) Microbiota changes at Family level.

(E–G) Antibiotics-pretreated mice (*n* = 4/group) were gavaged with fecal microbiota from mice treated with or without butyrate under steady conditions. (E) The diagram of the experiment design. (F) Representative flow cytometry plots of intestinal GzmB^+^ CD4^+^ T cells. (G) The Dot plots of intestinal GzmB^+^ CD4^+^ T cells.

(H–J) Antibiotics-pretreated mice (*n* = 4/group) were gavaged with fecal microbiota from mice treated with or without butyrate under activated conditions. (H) The diagram of the experiment design. (I) Representative flow cytometry plots of intestinal GzmB^+^ CD4^+^ T cells. (J) The Dot plots of intestinal GzmB^+^ CD4^+^ T cells.

All data are presented as mean ± SEM and are one representative of two independent experiments (E–J). (A, C, D, G, and I) Unpaired Student’s *t*-test; (B) weighted Bray-Curtis PCoA analysis; *p < 0.05; ns, not significance.

### HDAC inhibition and GPR43, but not GPR41 and GPR109a, mediate butyrate induction of GzmB in CD4^+^ T cells

**Figure 4.**
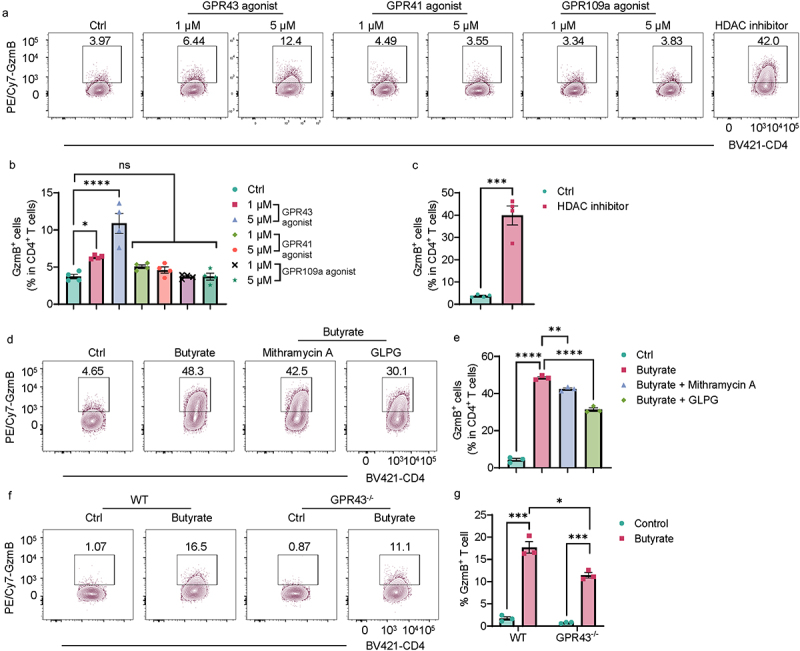
GPR43 and HDAC inhibitor mediate butyrate induction of GzmB in Th1 cells.

We next investigated how butyrate upregulates GzmB expression in CD4^+^ T cells. Since butyrate functions via binding receptors (i.e., GPR41, GPR43, and GPR109a) or/and inhibiting histone deacetylase (HDAC)^2^, we used a variety of agonists and antagonists/inhibitors for activating or inhibiting these pathways in CD4^+^ T cells treated with or without butyrate under Th1 conditions. We found that HDAC inhibitor, TSA, and GPR43 agonist, but not GPR41 agonist and GPR109a agonist, increased GzmB expression in CD4^+^ T cells (Figure 4A–C). Furthermore, GPR43 antagonists, GLPG 0974,^14^ and inhibitor of HDAC inhibition, Mithramycin A,^15^ suppressed butyrate induction of GzmB in CD4^+^ T cells (Figure 4D–E). Next, we treated splenic CD4^+^ T cells isolated from WT and GPR43^−/−^ mice, with or without butyrate under Th1 conditions. The levels of butyrate-induced GzmB expression in GPR43^−/−^ CD4^+^ T cells were lower than in butyrate-treated WT T cells (Figure 4F,G). These data suggest that HDAC inhibition and the GPR43 pathway are involved in the butyrate induction of GzmB in CD4^+^ T cells.

(A-E) Splenic CD4^+^ T cells were activated with anti-mCD3 and anti-mCD28 in the presence or absence of butyrate (500 µM) under Th1 conditions. (A–C) Cells (*n* = 4/group) were treated with or without GPR43 agonist, GPR41 agonist, GPR109a agonist, and HDAC inhibitor (TSA, 10 nM) for 5 days. (A) Representative flow cytometry plots of GzmB^+^ CD4^+^ T cells. (B,C) Quantification of GzmB^+^ CD4^+^ T cells. (D,E) Cells (*n* = 4/group) were treated with or without Mithramycin A (10 nM) and GLPG (10 nM) for 5 days. (D) Representative flow cytometry plots of GzmB^+^ CD4^+^ T cells. (E) Quantification of GzmB^+^ CD4^+^ T cells.

(F-G) WT and GPR43^−/−^ splenic CD4^+^ T cells were activated with anti-mCD3 and anti-mCD28 in the presence or absence of butyrate (500 µM) under Th1 conditions for 5 days. (F) Representative flow cytometry plots of GzmB^+^ CD4^+^ T cells. (G) Quantification of GzmB^+^ CD4^+^ T cells.

All data are presented as mean ± SEM and are one representative of three independent experiments. (B and E) one-way ANOVA with Dunnett’s multiple comparisons test; (C and G) unpaired Student’s *t*-test; *p < 0.05; ***p < 0.001; ****p < 0.0001; ns, not significance.

### Blimp1 is involved in the butyrate induction of GzmB in IL-10-producing Th1 cells.

**Figure 5.**
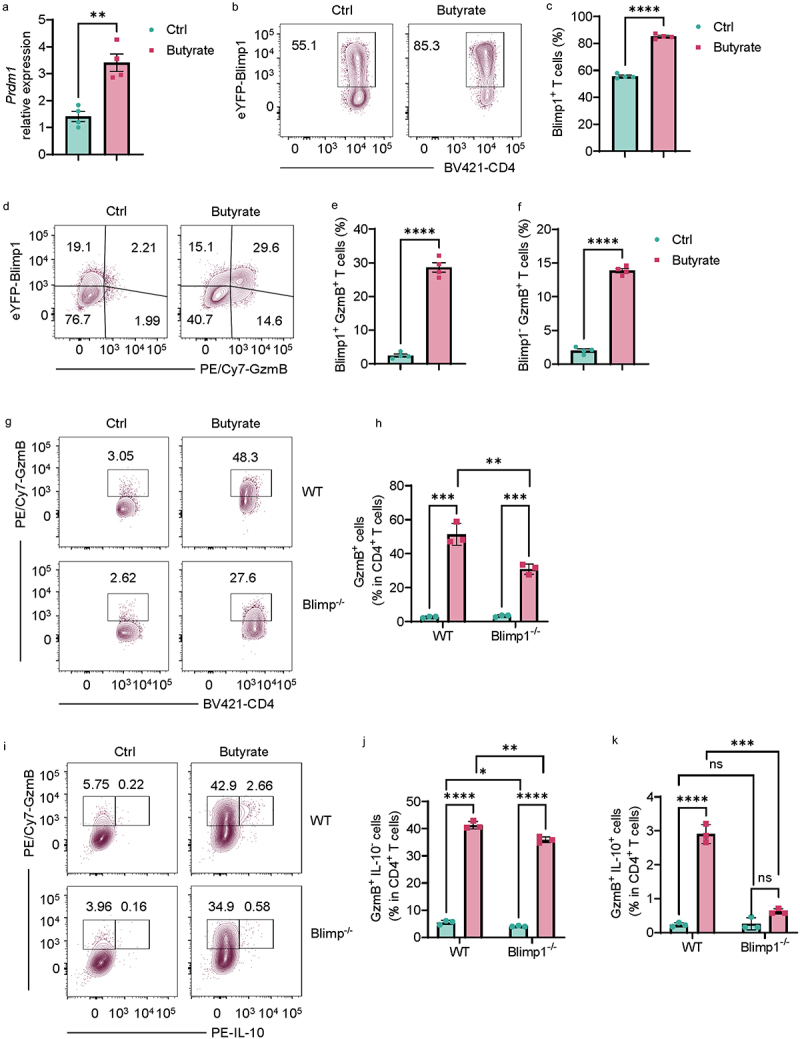
Blimp1 mediates butyrate induction of GzmB in Th1 cells.

Blimp1 has been reported to regulate GzmB expression in CD8^+^ T cells^16^ and mediate SCFAs induction of IL-10 in CD4^+^ T cells.^8^ We then investigated whether Blimp1 mediates butyrate induction of GzmB in Th1 cells. Consistent with our previous data,^13^ butyrate increased Blimp1 mRNA expression in CD4^+^ T cells under Th1 conditions (Figure 5A). Blimp1 protein level was also increased after butyrate treatment (Figure 5B,C). Furthermore, butyrate increased the population of Blimp1^+^ GzmB^+^ T cells and Blimp1^−^ GzmB^+^ T cells (Figure 5D–F), indicating Blimp1 might partially participate in butyrate induction of GzmB production in T cells. Next, we treated WT and Blimp-deficient CD4^+^ T cells with or without butyrate under Th1 conditions and found that butyrate induction of GzmB was compromised in Blimp1^−/−^ T cells compared to WT T cells (Figure 5G,H). We found that butyrate failed to induce GzmB production in IL-10-producing T cells under Th1 conditions (Figure 5I–k). These data indicate that Blimp1 is involved in the butyrate induction of GzmB in CD4^+^ T cells, especially IL-10-producing Th1 cells.

Splenic CD4^+^ T cells were activated with anti-mCD3 and anti-mCD28 in the presence or absence of butyrate (500 µM) under Th1 conditions.

(A) *Prdm1* mRNA expression in T cells on day 2 (*n* = 4/group).

(B) Representative flow cytometry plots of Blimp1-YFP^+^ CD4^+^ T cells on day 5.

(C) Quantification of Blimp1-YFP^+^CD4^+^ T cells on day 5 (*n* = 4/group).

(D) Representative flow cytometry plots of Blimp1-YFP^+^GzmB^+^ CD4^+^ T cells and Blimp1-YFP^−^GzmB^+^ CD4^+^ T cells on day 5 (*n* = 4/group).

(E) Quantification of Blimp1-YFP^+^GzmB^+^ CD4^+^ T cells on day 5 (*n* = 4/group).

(F) Quantification of Blimp1-YFP^−^GzmB^+^ CD4^+^ T cells on day 5 (*n* = 4/group).

(G) Representative flow cytometry plots of GzmB^+^ WT and Blimp1^−/−^ CD4^+^ T cells on day 5 (*n* = 3/group).

(H) Quantification of GzmB^+^ WT and Blimp1^−/−^ CD4^+^ T cells on day 5 (*n* = 3/group).

(I) Representative flow cytometry plots of GzmB^+^ IL-10^−^ and GzmB^+^ IL-10^+^ WT/ Blimp1^−/−^ CD4^+^ T cells on day 5 (*n* = 3/group).

(J) Quantification of GzmB^+^ IL-10^−^ WT/ Blimp1^−/−^ CD4^+^ T cells on day 5 (*n* = 3/group).

(K) Quantification of GzmB^+^ IL-10^+^ WT/ Blimp1^−/−^ CD4^+^ T cells on day 5 (*n* = 3/group).

All data are presented as mean ± SEM and are one representative of three independent experiments. Unpaired Student’s *t*-test; *p < 0.05; **p < 0.01; ***p < 0.001; ****p < 0.0001; ns, not significance.

### Glucose metabolism is involved in the butyrate induction of GzmB in Th1 cells.

**Figure 6.**
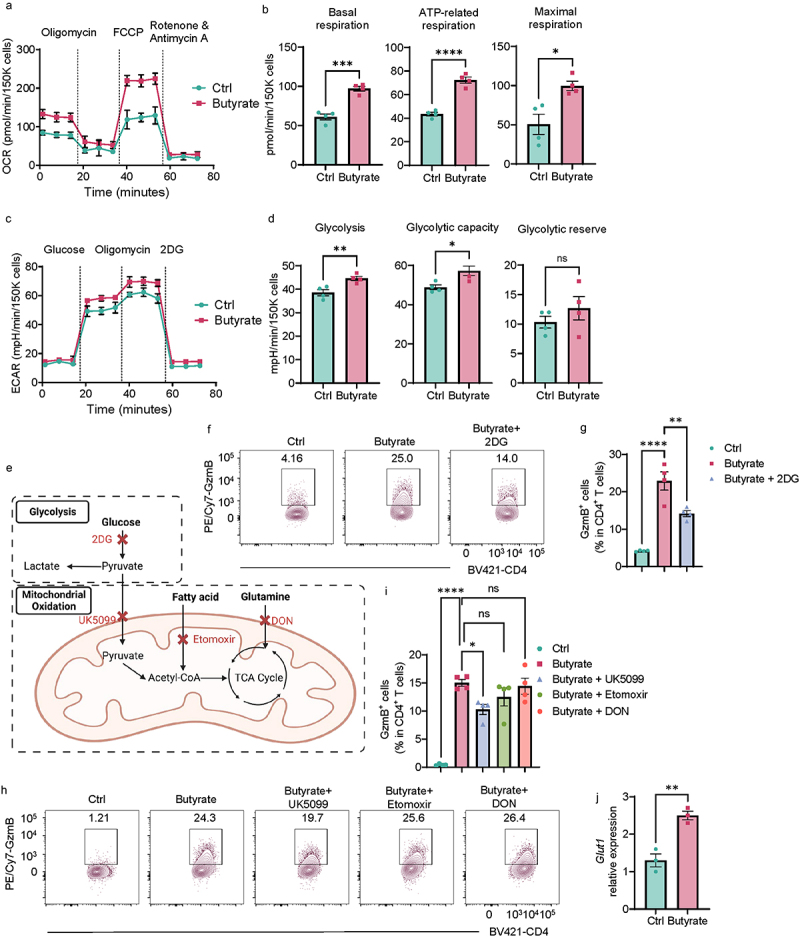
Glucose metabolism mediates butyrate induction of GzmB in Th1 cells.

Accumulating evidence indicates a crucial role of metabolic programs in maintaining T cell activation and functions.^17^ In addition, butyrate has been reported to affect cellular metabolic patterns in several cell types, including colonocytes, CD8^+^ T cells, and CD4^+^ T cells.^18–20^ We then investigated whether butyrate modulates Th1 cell metabolism, especially mitochondrial oxidation and glycolysis, to promote GzmB production. We activated CD4^+^ T cells with αCD3 mAb and αCD28 mAb under Th1 conditions in the presence or absence of butyrate for 24 h and measured the oxygen consumption rate (OCR), which is primarily attributed to mitochondrial oxidation, and the extracellular acidification rate (ECAR) that represents glycolysis, by using an extracellular flux Seahorse analyzer. As shown in Figure 6A,B, butyrate-treated T cells exhibited enhanced OCR with higher levels of basal respiration, ATP-linked respiration, and maximal respiration compared with controls. The levels of glycolysis, glycolytic capacity, and glycolytic reserve were also increased in butyrate-treated T cells (Figure 6C,D). These data suggested that butyrate increases both mitochondrial oxidation and glycolysis in Th1 cells.

(A–B) The parameters of mitochondrial respiration in control Th1 and butyrate-pretreated Th1 cells were measured by a Mito Stress Test Kit (*n* = 4/group). (A) The OCR profile. (B) Basal respiration; ATP-related respiration; Maximal respiration.

(C,D) Glycolysis-related parameters in control Th1 and butyrate-pretreated Th1 cells were measured by a Glycolysis Stress Test Kit (*n* = 4/group). (A) The ECAR profile. (B) Glycolysis; Glycolytic capacity; Glycolytic reserve.

(E) A diagram of different metabolic pathways contributing to glycolysis and mitochondrial oxidation.

(F,G) Splenic CD4^+^ T cells were activated with anti-CD3 (5 µg/mL) and anti-CD28 (2 µg/mL) antibodies in the presence or absence of butyrate (500 µM) with or without 2-DG under Th1 conditions for 5 days. (F) Representative flow cytometer plots of GzmB^+^ CD4^+^ T cells. (G) Quantification of GzmB^+^ CD4^+^ T cells.

(H–J) Splenic CD4^+^ T cells were activated with anti-CD3 (5 µg/mL) and anti-CD28 (2 µg/mL) antibodies in the presence or absence of butyrate (500 µM) with or without UK5099 (30 µM)/etomoxir (50 µM)/DON (1 µM) under Th1 conditions for 5 days. (H) Representative flow cytometer plots of GzmB^+^ CD4^+^ T cells. (I) Quantification of GzmB^+^ CD4^+^ T cells. (J) *Glut1* mRNA expression in T cells on day 2 (*n* = 3/group).

All data are presented as mean ± SEM and are one representative of three independent experiments. (B, D, and J) Unpaired Student’s *t*-test; (G and I) one-way ANOVA with Dunnett’s multiple comparisons test; *p < 0.05; **p < 0.01; ***p < 0.001; ****p < 0.0001; ns, not significance.

To investigate whether enhanced mitochondrial oxidation and glycolysis contribute to GzmB in Th1 cells induced by butyrate, we used a variety of inhibitors to inhibit different pathways in glycolysis and mitochondrial oxidation (Figure 6E). First, we treated Th1 cells with 2-Deoxy-D-glucose (2DG), a synthetic glucose analog, which blocks glucose metabolism, including glycolysis and pyruvate oxidation (Figure 6E). We found that 2DG suppressed GzmB production induced by butyrate in Th1 cells (Figure 6F,G). Glucose, Fatty acids, and glutamine are the major resources of mitochondrial oxidation through pyruvate oxidation, β-oxidation, and glutaminolysis, respectively. Therefore, we used UK5099, Etomoxir, and Don to inhibit these three oxidation pathways (Figure 6E). We found that only UK5099 decreased GzmB expression induced by butyrate in Th1 cells (Figure 6H,I), indicating that glucose-derived pyruvate oxidation mediates butyrate induction of GzmB. In addition, we found that butyrate increased Th1 cell expression of glucose transporter 1 (Glut1) (Figure 6J), a major glucose transporter in T cells.^21^ Taken together, glucose metabolism is critical in regulating butyrate induction of GzmB in Th1 cells, probably through upregulating glucose transport.

### IKZF3 and NR2F6 mediate butyrate induction of GzmB in Th1 cells.

**Figure 7.**
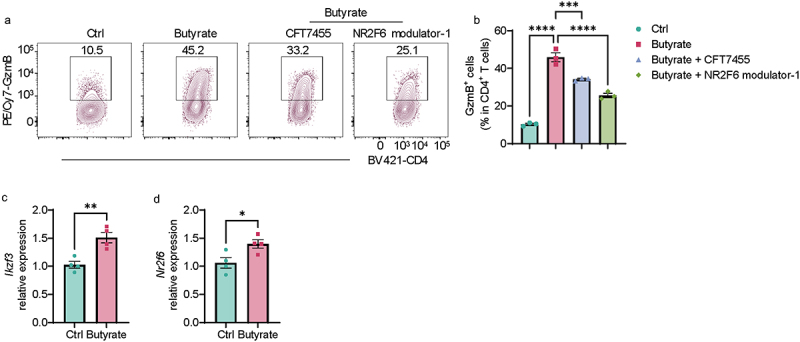
IKZF3 and NR2F6 regulate butyrate induction of GzmB in Th1 cells.

To investigate whether other transcription factors, in addition to Blimp1, mediate butyrate induction of GzmB in Th1 cells, we used the JASPAE2022 to predict the potential transcription factors regulating GzmB expression (Supplementary Figure S5A). We re-analyzed RNA-seq data and found that butyrate differentially regulated mRNA levels of the predicted transcription factors (Supplementary Figure S5A). After searching, only three commercial inhibitors or antagonists were found, including IKZF3 degrader CFT7455, NR2F6 modulator-1, and NR4A1 antagonist DIM-c-pPhco2ME (Supplementary Figure S5A). We found that IKZF3 degrader CFT7455 and NR2F6 modulator-1, but not NR4A1 antagonist DIM-c-pPhco2ME, suppressed butyrate-induction of GzmB in Th1 cells (Figure 7A,B, and Supplementary Figures S5B,C). Furthermore, we confirmed that butyrate promoted *Ikzf3* and *Nr2f6* expression in Th1 cells by qRT-PCR (Figure 7C,D). These data suggested that IKZF3 and NR2F6 are involved in the butyrate induction of GzmB in Th1 cells.

Splenic CD4^+^ T cells were activated with anti-CD3 (5 µg/mL) and anti-CD28 (2 µg/mL) antibodies in the presence or absence of butyrate (500 µM) with or without CFT7455 (1 nM) or NR2F6 modulator-1 (10 µM) under Th1 conditions.

(A) Representative flow cytometer plots of GzmB^+^ CD4^+^ T cells on day 5.

(B) Quantification of GzmB^+^ CD4^+^ T cells on day 5.

(C) *Ikzf3* mRNA expression on day 2.

(D) *Nr2f6* mRNA expression on day 2.

## Discussion

It has been established that gut microbiota are critical in regulating intestinal immune responses and diseases, where microbiota-derived metabolites play an important role.^2^ As one of the most investigated metabolites, SCFAs exert different effects on various immune cells. Here, we demonstrated that butyrate increases GzmB expression in tolerogenic IL-10-producing Th1 cells, thereby contributing to maintaining intestinal homeostasis. This report expands our knowledge of the interaction among gut microbiota, Th1 cell responses, and intestinal diseases.

Th1 cells are considered an important contributor to intestinal inflammation. However, IL-10-producing Th1 cells are less pathogenic, as autocrine IL-10 limits excessive Th1 responses.^22^ Our previous study found that butyrate transforms IL-10^−^ Th1 cells into IL-10-producing Th1 cells.^8^ This report found that butyrate promotes GzmB expression in both IL-10^−^ Th1 cells and IL-10^+^ Th1 cells. Interestingly, CD4^+^ T cells expressing IFN-γ^high^, IL-10^high^, and GzmB^high^, are the most increased population induced by butyrate. Blimp1, which has been reported to mediate butyrate induction of IL-10 in Th1 cells, is only involved in butyrate induction of GzmB in IL-10-producing Th1 cells, but not IL-10^−^ Th1 cells. We proposed that IL-10 and GzmB double-positive Th1 cells play a critical role in butyrate protection against colitis. However, further experiments are necessary to validate this hypothesis. If proven, it could enable the prediction of individual responsiveness to butyrate treatment by assessing butyrate responsiveness using patients’ blood *in vitro*, akin to antimicrobial susceptibility testing.

GF mice exhibited a decreased population of GzmB-producing CD4^+^ T cells in the intestine, suggesting that gut microbiota is critical in inducing GzmB-producing CD4^+^ T cells. Butyrate, the microbiota-derived metabolites, increased the GzmB expression in CD4^+^ T cells in SPF mice, indicating that microbiota promotes T cell production of GzmB at least partially through butyrate. Although butyrate affected gut microbiota composition in mice, the butyrate-altered microbiota did not increase intestinal CD4^+^ T cell expression of GzmB compared to the control microbiota. Although these data may suggest that butyrate directly acts on T cells to induce GzmB, we have not yet ascertained whether the induction of GzmB by butyrate is microbiota-dependent or not, as we did not investigate whether butyrate could induce GzmB-producing T cells in GF mice. If this induction does not occur in GF mice, it would suggest that the gut microbiota is indispensable for the butyrate-mediated induction of GzmB. Then, further investigation should be done to identify which specific bacteria species contributing to this process.

CD8^+^ T cell and natural killer cell production of GzmB has been reported to mediate intestinal infection and tumors through cytotoxic activity. However, the role of GzmB-producing CD4^+^ T cells in colitis is controversial as both pro- and anti-inflammatory effects were reported. The cytotoxic activity of GzmB-producing CD4^+^ T cells was found to promote intestinal inflammation,^23^ while a recent study has reported that CD4^+^ T cell-expressed GzmB suppresses intestinal inflammation through regulating Th17 responses,^12^ which reveals that GzmB regulation of immune responses is not limited to cytotoxic activity. These conflicting findings suggest the role of GzmB-producing CD4^+^ T cells in colitis, and the factors affecting the functions of these cells need more comprehensive investigation. Our finding that GzmB-deficient Th1 cells induce more severe colitis in immune-deficient mice than WT Th1 cells suggests that GzmB is also involved in Th1 responses to regulate intestinal homeostasis. Furthermore, butyrate-pretreated GzmB^−/−^ Th1 cells induce more severe colitis than butyrate-pre-treated WT Th1 cells, indicating that GzmB mediates butyrate regulation of intestinal Th1 responses. In this study, we found Th1 cells suppressed T cell death, which might contribute to exaggerated T cell responses. The capacity of inhibiting T cell death was similar between WT and GzmB-deficient Th1 cells, which indicates that cytotoxic activity might not be the most important function of Th1-expressed GzmB in regulating T cell-mediated diseases. However, these data are preliminary, further investigation is required. In addition, we did not perform the cytotoxic activity of IL-10 and GzmB double-positive cells as we do not have the reporter mice. Interestingly, GzmB^−/−^ Th1 cells showed decreased suppressive function toward T cells, although the mechanisms involved were not investigated in this study.

In this study, we found that butyrate promotes IL-10 and GzmB double-positive cells. However, we did not perform the cytotoxic activity of IL-10 and GzmB double-positive cells as we do not have the reporter mice. Interestingly, oral butyrate treatment increases intestinal Th1 expression of GzmB under both steady and activated conditions, highlighting the translational potential of butyrate in treating intestinal inflammation through the upregulation of GzmB expression in Th1 cells. To further confirm the effect of CD4^+^ T cell production of GzmB on colitis, it is essential to introduce CD4^+^ T cell-specific GzmB knockout mice and utilize additional colitis models, such as the DSS model, with these mice.

Blimp1 is the transcription factor of IL-10 and participates in SCFAs induction of IL-10 in Th1 cells.^8^ Our study unveils its additional role in butyrate-induced GzmB production in Th1 cells. However, butyrate could still promote GzmB production in Blimp1-deficient Th1 cells, indicating that Blimp1 is not the sole factor affecting this process. Our investigation revealed that IKZF3 and NR2F6, two additional transcription factors, also play a pivotal role in mediating this intricate process. Interestingly, we found that Blimp1 is the key factor mediating GzmB production in IL-10-producing Th1 cells. This conclusion is supported by the observation that butyrate failed to induce IL-10^+^ GzmB^+^ T cells in Blimp1-deficient T cells. However, further investigation is warranted to determine whether Blimp1 influences GzmB expression directly or through its impact on IL-10.

Both microbiome and cell metabolism are important factors in regulating immune cell responses. Although butyrate altered the gut microbiome in mice, the influence of butyrate on the gut microbiome did not directly mediate GzmB induction in T cells. Instead, we found that glucose metabolism, including glycolysis and glucose-derived pyruvate oxidation, is critical to butyrate induction of GzmB. Despite this insight, specific genes within the glucose metabolism pathway controlled by butyrate remain unidentified. In addition, the association between Blimp1 and glucose metabolism in this context should also be investigated in future studies.

## Materials and methods

### Mice

C57BL/6J wild-type (WT) mice, B6.129-Prdm1tm1Clme/J (*Prdm*^fl/fl^) mice, B6.Cg-Tg(Cd4-cre)1Cwi/BfluJ (*Cd4*^cre^) mice, and B6.Cg-Tg(Prdm1-EYFP)1Mnz/J mice, B6.SJL-*Ptprc*^*a*^ *Pepc*^*b*^*/*BoyJ (CD45.1) mice were obtained from Jackson Laboratory. GzmB^−/−^ spleens and paired WT spleens were kindly provided by Dr. Danyvid Olivares-Villagómez, Vanderbilt University Medical Center. *Prdm1*^fl/fl^ mice were crossed to *Cd4*^cre^ to generate *Cd4*^cre^ *Prdm*^fl/fl^ mice. These mice were maintained on a 12-hour-light/dark cycle with a temperature of 20–26°C and 30–70% humidity in the specific pathogen-free (SPF) animal facility of the University of the Texas Medical Branch. Germ-free (GF) mice were maintained in the Gnotobiotics Facility and paired SPF mice were maintained in the barrier housing of the Center for Comparative Medicine at Northwestern University. Before use, all the mice experiments were reviewed and approved by the Institutional Animal Care and Use Committee of the University of the Texas Medical Branch or the Animal Care and Use Committee of Northwestern University. All the mice used in this study were sex-matched and 6–12 weeks of age.

### Mice treatment

Mice were treated with butyrate (200 mM) in drinking water for two weeks. For T cell activation, mice were intraperitoneally given an anti-CD3 antibody (0.75 mg/kg) twice, two days and four hours before being killed.

### CD4^+^ cell transfer model

Sorted WT or GzmB^−/−^ CD4^+^ CD45Rb^hi^ T cells (1 × 10^5^ cells/mouse) were intravenously transferred to *Rag*^−/−^ mice. Mice were sacrificed five weeks later.

Butyrate-treated Th1 cells or control Th1 cells from WT or GzmB^−/−^ mice (1 × 10^6^ cells/mouse) were intravenously transferred to *Rag*^−/−^ mice. Mice were sacrificed five weeks later.

### Fecal microbiota transplantation

Feces were freshly collected from WT mice pretreated with or without butyrate (four weeks) and resuspended in the 1 × PBS supplemented with L-cysteine hydrochloride (0.05%, w/v). After homologation, large particles were removed from fecal suspension through centrifugation and strainers. Fecal bacteria suspension was then orally transferred to antibiotics-pretreated mice via gavage three times on days 0, 7, and 14. Recipients were killed four weeks post-first gavage.

For antibiotics treatment, mice were orally administered an antibiotic cocktail of 1 g/L Ampicillin, 1 g/L Kanamycin, 0.5 g/L Vancomycin, and 1 g/L Metronidazole in drinking water for two weeks.

### Primary mouse CD4^+^ T cell cultures

Splenic mouse CD4^+^ T cells (2 × 10^5^ cells/ml) were suspended in the complete RPMI 1640 media containing penicillin-streptomycin, L-glutamine, sodium pyruvate, β-mercaptoethanol, and 10% FBS with anti-mouse CD28 (2 µg/ml). The cell suspension was then seeded into the anti-mouse CD3 (5 µg/ml) pre-coated plates and cultured in a humidified incubator with 5% CO_2_ at 37°C.

### Hematoxylin/Eosin (H&E) staining and histopathological evaluation

Mouse colon samples were cut longitudinally, rolled into the Swiss roll, fixed in 10% neutral-buffered formalin for 24 hours, and embedded in paraffin. Paraffin-embedded samples were cut into 5-µm sections. After de-waxing and hydration, sections were stained with hematoxylin followed by eosin using an H&E staining kit (ab245880, Abcam).

Colon sections were imaged by Leica DM1000 with IC50, and the colitis severity was evaluated under the specified criteria. The pathology scores were calculated from six parameters for a maximum score of 15: lamina propria inflammation (normal, 0; mild, 1; moderate, 2; severe, 3), goblet cell loss (normal, 0; mild, 1; moderate, 2; severe, 3), abnormal crypt (normal, 0; hyperplastic, 1; disorganization, 2; crypt loss, 3), crypt abscesses (absent, 0; present, 1), mucosal erosion and ulceration (normal, 0; mild, 1; moderate, 2; severe, 3), and submucosal change (none, 0; submucosa, 1; transmural, 2).

### Enzyme-linked immunosorbent assay (ELISA)

Two colonic punch biopsies were cultured in complete RPMI 1640 medium for 24 hours. Supernatants were then collected for analysis of TNF-α secretion using ELISA MAX™ Deluxe Sets (Biolegend). 96-well ELISA plates were pre-coated with the capture antibodies overnight at 4°C. After washing the plates, supernatants were incubated in the plates for 2 hours at room temperature. Then, detection antibodies were added for incubation at room temperature for 1 hour. Streptavidin conjugated with horseradish peroxidase was then incubated in the plates for another 1 hour. After adding the substrate, the absorbance was measured at 450 nm using BioTek Gene5.

### Intestinal lamina propria cell isolation

Mouse colons were cut into 1-cm pieces and put into 50-ml centrifuge tubes containing cold PBS. After washing, colon pieces were incubated with 0.5 Mm ETDA for 40 minutes. After discarding supernatants, tissues were washed and incubated with 0.5 mg/ml Collagenase IV and 2 µg/ml DNAse in the gentleMACS^TM^ tubes on the gentleMACS^TM^ Dissociator. Finally, cell supernatants were collected by passing through the 100-µm cell strainer, and lamina propria cells were isolated using 40%/70% Percoll.

### Flow cytometry

Intestinal lamina propria cells or splenic CD4^+^ T cells were activated with Phorbol-12-myristate 13-acetate (20 ng/ml, Millipore Sigma) and ionomycin (750 ng/ml, Thermo Fisher Scientific) for 2 hours, followed by incubation with Brefeldin A (1 µl/ml, BD Biosciences) for 3 hours. Cells were collected for incubation with anti-CD16/32 (1: 40, Biolegend) to block the Fc, followed by staining with live dye using LIVE/DEAD™ Fixable Near-IR Dead Cell Stain Kit (Invitrogen) and surface staining with anti-CD4 (1: 40, Biolegend). After 30 minutes, cells were permeabilized using Foxp3/Transcription Factor Fixation/Permeabilization set (Thermo Fisher Scientific) or Intracellular Fixation & Permeabilization Buffer Set (Thermo Fisher Scientific). Subsequently, cells were stained with anti-IFNγ (1: 20, Biolegend), anti-GzmA (1: 40, Biolegend), anti-GzmB (1: 40, Biolegend), anti-IL-10 (1: 40, Biolegend) for 30 minutes. Finally, cells were collected by BD FACSymphony A5 SE and BD FACS Diva software and analyzed by FlowJo. For high-dimensional data clustering, after sample cleaning, concentration, and dimensionality reduction (tSEN), clustering was done using FlowSOM.

### CellTracer and viability

CD45.1 naïve CD4^+^ T cells (4,000 cells/well) were stained with CellTracer Violet and then cocultured with WT or GzmB-deficient Th1 cells (4,000 cells/well) in the presence of irradiated antigen-presenting cells (16,000 cells/well) and anti-CD3 antibody (5 µg/mL) in a 96-well plate. After 60 hours, cells were collected and stained with anti-CD4 (1: 40, Biolegend) and anti-CD45.1 (1: 40, BD Biosciences) for 15 minutes. After washing, cells were stained with 7-AAD and then collected by BD FACSymphony A5 SE and BD FACS Diva software and analyzed by FlowJo.

### Mouse CD4^+^ T cell isolation

Mouse spleens were ground and suspended in RPMI 1640. Splenic cells were collected by passing through a 100-µm cell strainer, and red blood cells were lysed with Tris-NH4CL. After incubating with anti-mouse CD4 magnetic particles (BD Biosciences) for 30 minutes at 4°C, cells were isolated using the Cell Separation Magnet.

### Mouse CD4^+^ T cell activation, differentiation, and treatment

Splenic CD4^+^ T cells were activated with anti-mCD3 (5 µg/ml) and anti-mCD28 mAb (2 µg/ml) under neutral conditions and Th1 polarization conditions (10 ng/ml IL-12). CD4^+^ T cells were activated in the presence or absence of butyrate (250 and 500 µM), GPR41 agonist (AR420626), GPR43 agonist (371725), GPR109a agonist (MK1903), GPR43 antagonists (GLPG, 10 nM), HDACi inhibitor (Mithramycin A, 10 nM), 2DG, UK5099, Etomoxir, DON, IKZF3 degrader (CFT7455, 1 nM), NF2F6 modulator-1 (10 µM), and NR4A1 antagonist (DIM-c-pPhco2ME, 0.1 nM) under the indicated conditions.

### RNA sequencing

Mouse splenic CD4^+^ T cells were activated with anti-mCD3 (5 µg/ml) and anti-mCD28 mAb (2 µg/ml) under Th1 conditions and cultured with or without butyrate (500 µM). Cells were collected after two days, and total RNA was extracted. All the RNA samples passed the qualifying examination. Samples were then processed by library construction at Novogene using a NEBNext^@^ Ultra RNA Library Prep Kit for Illumia^@^. All the analyses were performed using NovoSmart.

### 16s rRNA sequencing

WT mice were orally treated with or without butyrate (200 mM) for four weeks. Feces were collected and DNA was extracted using a QIAAMP PowerFecal DNA kit (Qiagen). DNA was amplified using 16S rRNA V3-V4 region primers, and sequencing was performed by an Illumina MiSeq Instrument. The alpha diversity was calculated using Shannon entropy (OTU level) and the beta diversity was determined by weighted Bray-Curtis dissimilarity matrices. Bar charts of the relative abundance were created by Atima2, developed by Center for Metagenomics and Microbiome Research of Baylor College of Medicine, and then modified by Graphpad Prism 10,

### Quantitative real-time PCR

Total RNA was extracted by Trizol, and reverse transcription was performed using Quanta Bio qScript^tm^ cDNA Synthesis Kit. Finally, gene expressions were analyzed by quantitative real-time PCR using iTaq Universal SYBR@ Green Supermix. Primers used in this study were listed as follows. *Gzmb* forward: CAGGAGAAGACCCAGCAAGTCA; *Gzmb* reverse: CTCACAGCTCTAGTCCTCTTGG; *Prdm1* forward: CTCAACACTCTCATGTAAGAGGC; *Prdm1* reverse: AGCATGACCTGACATTGACACC; *Ikzf3* forward: GCCGTACAAGTGTGAGTTCTGC; *Ikzf3* reverse: CTCGGCTTTGATGTGTCTTGCC; *Nr2f6* forward: GTCCAGTGGAAAGCATTACGGC; *Nr2f6* reverse: GCTGATCAATCTGACAGTCACGG; *Glut1* forward: GCTTCTCCAACTGGACCTCAAAC; *Glut1* reverse: ACGAGGAGCACCGTGAAGATGA.

### Metabolic profile analysis

Mouse splenic CD4^+^ T cells were activated with anti-mCD3 (5 µg/ml) and anti-mCD28 mAb (2 µg/ml) and cultured with or without butyrate (500 µM) under Th1 conditions for 48 hours. The oxygen consumption rate (OCR) and extracellular acidification rate (ECAR) were analyzed by the Seahorse XF Cell Mito Stress Test Kit and the Seahorse XF Glycolysis Stress Test Kit (Aligent).

### Quantification and statistical analysis

Data were analyzed by two-way Student’s *t*-test or Mann–Whitney U test or one-way ANOVA test using GraphPad Prism 10. All data are presented as mean ± SEM, and the *p*-value <0.05 was considered statistically significant.

## Supplementary Material

Supplementary Figures revision 041824.docx

## Data Availability

RNA sequencing data in this study is available in the GEO database under the accession number GSE139631. 16s rRNA sequencing data has been deposited in the SRA database under the accession number PRJNA1059631.
