# Supplementary material for: Microbial metabolite butyrate modulates granzyme B in tolerogenic IL-10 producing Th1 cells to regulate intestinal inflammation
Source: Gut Microbes. 2024 Jun 6;16(1):2363020. doi: 10.1080/19490976.2024.2363020 (PMC11164233; doi:10.1080/19490976.2024.2363020)
Supplement: Supplementary Figures revision 041824.docx [file KGMI_A_2363020_SM0632.docx]

**
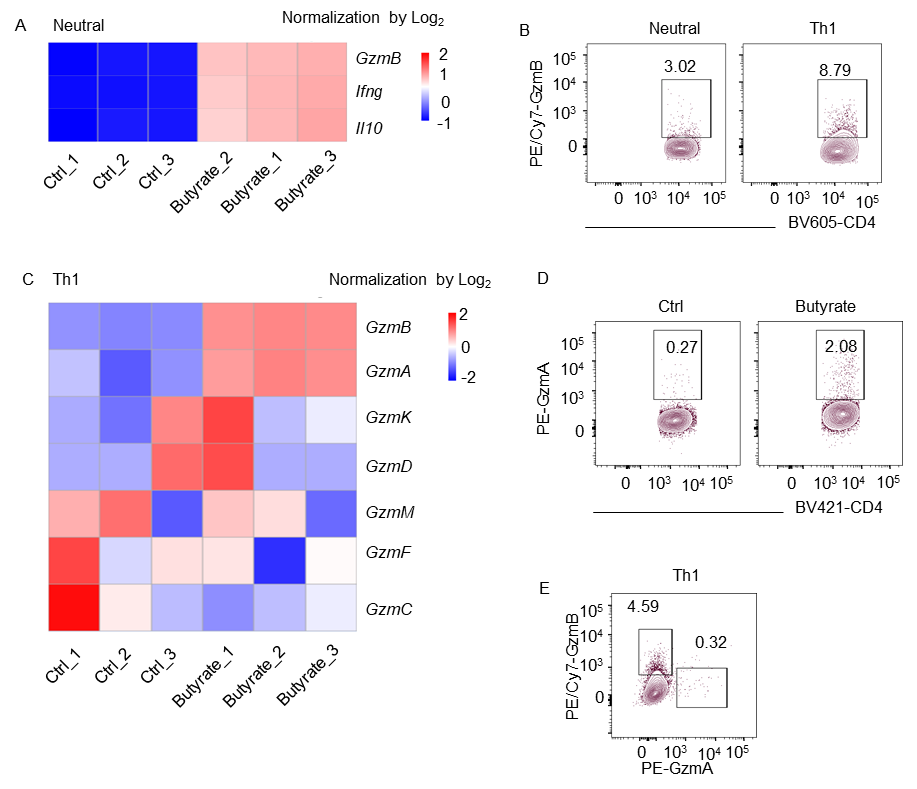
Supplementary Figures**

**Supplementary Figure 1. Th1 cells express a higher expression of GzmB than T cells under neutral conditions.**

(A) Splenic CD4^+^ T cells were activated with anti-mCD3 and anti-mCD28 in the presence or absence of butyrate (500 µM) under neutral conditions. Cells were collected after 2 days for RNA-Seq (n = 3/group). Heatmap of *Ifng*, *Il10*, and *Gzmb* between two groups. Arbitrary units. (B) Splenic CD4^+^ T cells were activated with anti-mCD3 and anti-mCD28 in the presence or absence of butyrate (500 µM) under neutral or Th1 conditions for 5 days. Representative flow cytometry plots of GzmB^+^ CD4^+^ T cells. (C) Splenic CD4^+^ T cells were activated with anti-mCD3 and anti-mCD28 in the presence or absence of butyrate (500 µM) under Th1 conditions. Cells were collected after 2 days for RNA-Seq (n = 3/group). Heatmap of gramzymes genes between two groups. (D-E) Splenic CD4^+^ T cells were activated with anti-mCD3 and anti-mCD28 in the presence or absence of butyrate (500 µM) under neutral or Th1 conditions for 5 days. (D) Representative flow cytometry plots of GzmA^+^ CD4^+^ T cells. (E) Representative flow cytometry plots of GzmA^+^ CD4^+^ T cells and GzmB^+^ CD4^+^ T cells.


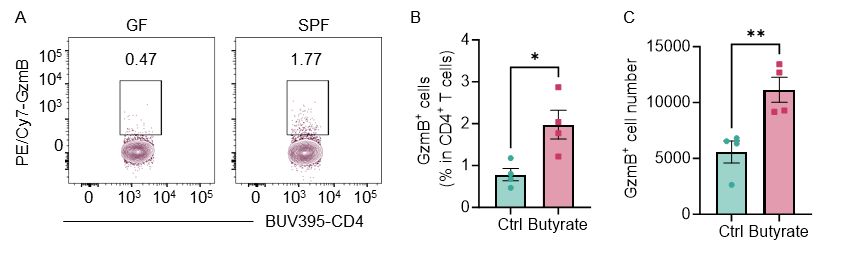


**Supplementary Figure 2. GzmB-producing CD4^+^ T cells are decreased in the intestinal of germ-free mice.**

WT and germ-free (GF) mice were killed. (A) Representative flow cytometry plots of GzmB^+^ CD4^+^ T cells in the colon. (B) Quantification of GzmB^+^ CD4^+^ T cells in the colon. (C) The absolute number of GzmB^+^ CD4^+^ T cells in the colon.

All data are presented as mean ± SEM. unpaired Student’s *t*-test; **p* < 0.05, ***p* < 0.01.

**Supplementary Figure 3. The absolute number of CD4^+^ T cells in the colon of *Rag1*^-/-^ mice receiving WT or GzmB^-/-^ T cells.**

CD4^+^ CD45Rb^hi^ T cells (1 × 10^5^ cells/ mouse) from WT mice or GzmB^-/-^ mice were intravenously transferred to *Rag*^-/-^ mice (n = 5/group). Mice were sacrificed five weeks later. The absolute number of CD4^+^ T cells in the colon.

All data are presented as mean ± SEM and are one representative of two independent experiments. unpaired Student’s *t-test*; ns, not significance.


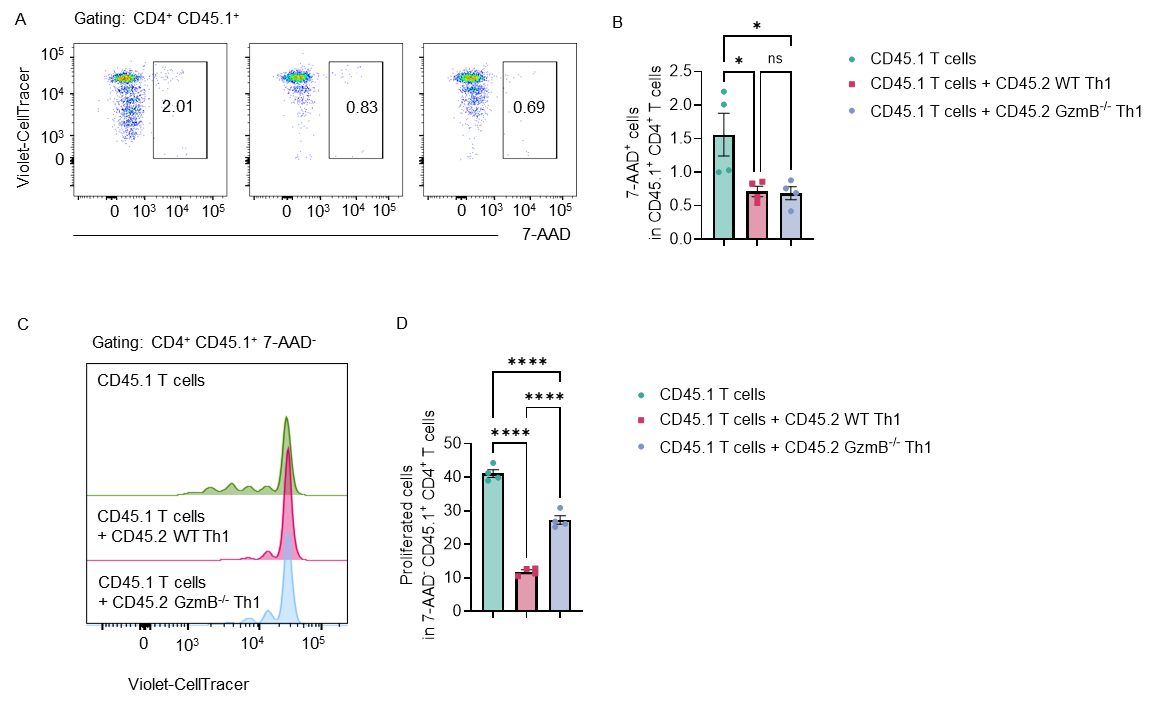


**Supplementary Figure 4. GzmB^-/-^ Th1 cells inhibit naïve CD4^+^ T cell proliferation.**

CD45.1 naïve CD4^+^ T cells (n = 4/group) were stained with CellTracer Violet and then cocultured with WT or GzmB-deficient Th1 cells (n = 4/group) in the presence of irradiated antigen-presenting cells and anti-CD3 antibody (5 µg/mL) in a 96-well plate. (A) Representative flow cytometry plots of CellTracer and 7-AAD. (B) The quantification analysis of 7-AAD^+^ cells in CD45.1^+^ CD4^+^ T cells. (C) Representative flow cytometry histogram of CellTracer. (D) The quantification of proliferated cells in 7AAD^-^ CD45.1^+^ CD4^+^ T cells.

All data are presented as mean ± SEM. One-way ANOVA with Dunnett's multiple comparisons test; *****p* < 0.0001.


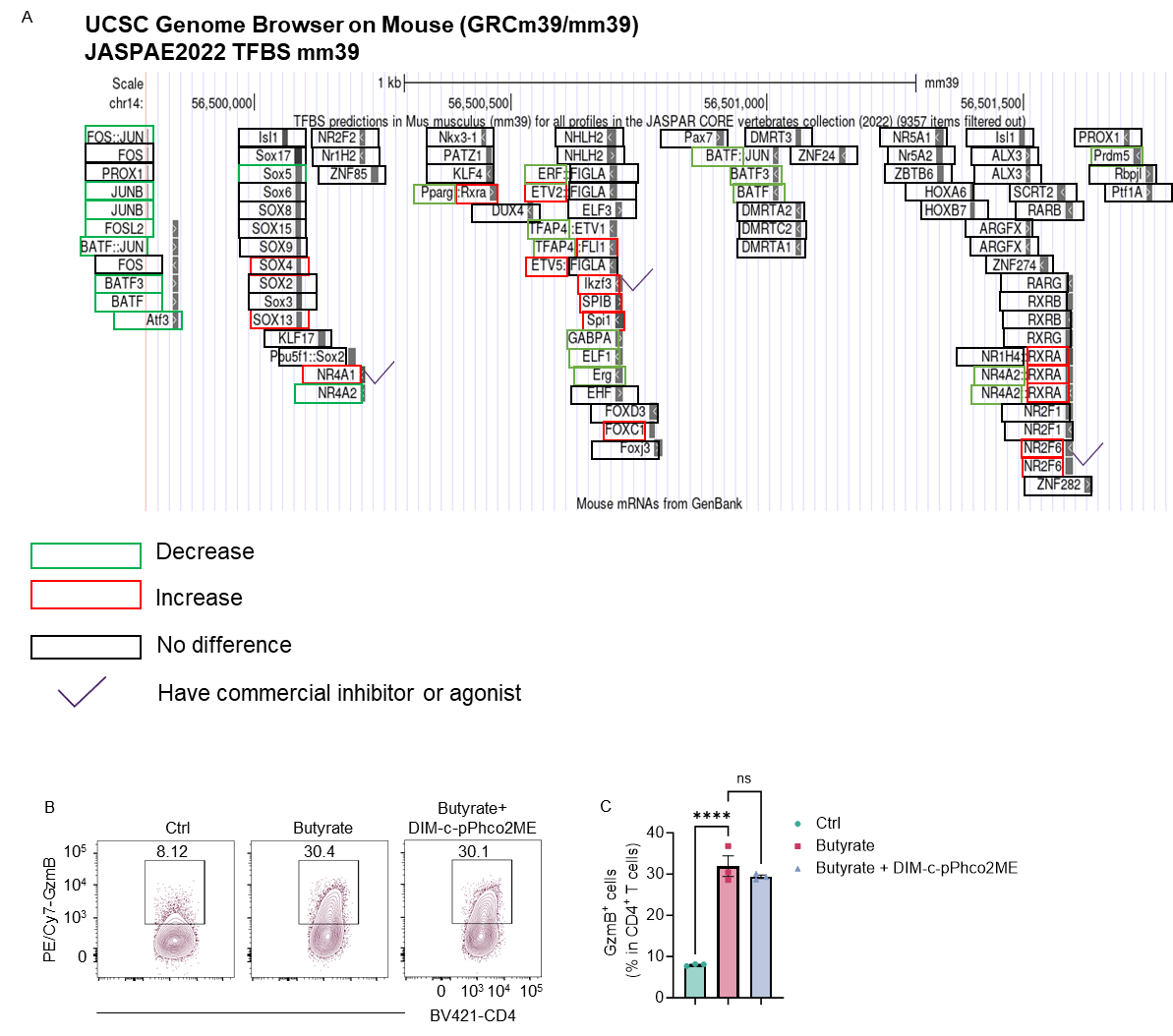


**Supplementary Figure 5. Potential transcription factors regulating GzmB expression.**

(A) Potential transcription factors regulating GzmB expression by JASPAW2022 TFBS mm39 with butyrate changed expression by RNA-seq.

(B-C) Splenic CD4^+^ T cells were activated with anti-mCD3 (5 µg/ml) and anti-mCD28 mAb (2 µg/ml) in the presence or absence of butyrate (500 µM) and/or NR4A1 antagonist (DIM-c-pPhco2ME, 0.1 nM) under Th1 polarization conditions for 5 days. (B) Representative flow cytometry plots of GzmB^+^ CD4^+^ T cells. (C) Quantification of GzmB^+^ CD4^+^ T cells.

All data are presented as mean ± SEM and are one representative of three independent experiments (B-C). One-way ANOVA with Dunnett's multiple comparisons test; *****p* < 0.0001; ns, not significance.
